# *SlbHLH22*-Induced Hypertrophy Development Is Related to the Salt Stress Response of the *GTgamma* Gene in Tomatoes

**DOI:** 10.3390/metabo13121195

**Published:** 2023-12-11

**Authors:** Baolu Cui, Min Yu, Jiaojiao Bai, Zhiguo Zhu

**Affiliations:** 1 College of Pharmacy and Life Sciences, Jiujiang University, Jiujiang 332005, China; 6090111@jju.edu.cn (B.C.); yuminyumin@whu.edu.cn (M.Y.); 2 College of Biological Sciences and Agriculture, Qiannan Normal University for Nationalities, Duyun 558000, China

**Keywords:** *SlbHLH22*-induced hypertrophy, metabolome, transcriptome, *GTgamma* gene, salt stress, *Solanum lycopersicum*

## Abstract

Hypertrophy development induced by the overexpression of *SlbHLH22* (also called *SlUPA-like*) was susceptible to *Xanthomonas* in tomatoes. Transcriptome and metabolome analyses were performed on the hypertrophy leaves of a *SlbHLH22*-overexpressed line (OE) and wild type (WT) to investigate the molecular mechanism. Metabolome analysis revealed that six key metabolites were over-accumulated in the OE, including Acetylserine/O-Acetyl-L-serine, Glucono-1,5-lactone, Gluconate, 2-Oxoglutarate, and Loganate, implying that the OE plants increased salt or oxidant resistance under normal growth conditions. The RNA-seq analysis showed the changed expressions of downstream genes involved in high-energy consumption, photosynthesis, and transcription regulation in OE lines, and we hypothesized that these biological processes were related to the GTgamma subfamily of trihelix factors. The RT-PCR results showed that the expressions of the *GTgamma* genes in tomatoes, i.e., *SlGT*-*7* and *SlGT*-*36*, were suppressed in the hypertrophy development. The expression of the *GTgamma* gene was downregulated by salinity, indicating a coordinated role of GTgamma in hypertrophy development and salt stress. Further research showed that both *SlGT*-*7* and *SlGT*-*36* were highly expressed in leaves and could be significantly induced by abscisic acid (ABA). The GTgamma protein had a putative phosphorylation site at S^96^. These results suggested GTgamma’s role in hypertrophy development by increasing the salt resistance.

## 1. Introduction

*Xanthomonas* causes a broad disease in crop cultivars, such as spot disease. To overcome plant defense, *Xanthomonas* delivers transcription activator-like effectors (TALes) into host cells to suppress immune responses [1]. AvrBs3, one of the TALe families, induces cell enlargement in the host leaf by directly activating a master regulator of cell size, i.e., *UPA20*, a bHLH family gene [2,3]. We also found that *SlUPA-like* (the orthology of *UPA20*, also called *SlbHLH22*) overexpression caused severe hypertrophy and facilitated the infection of *Xanthomonas* in tomato leaves. The experimental evidence proved that the Gibberellin (GA) response was upregulated and that the jasmonic acid (JA) response was downregulated in *SlUPA-like* overexpressed lines (OEs) [4]. Additionally, the mature leaves of OEs curled upward and wilted under normal conditions, and the total chlorophyll decreased remarkably [4]. These phenotypes implied that other factors might be involved in the developmental malformation of OE plants.

Previous reports proved that altering plant development with trihelix factors contributes to pathogen susceptibility or resistance. *GhGT-3b* was strongly induced by *Verticillium dahlia* and the heterologous expression of *GhGT-3b* in *Arabidopsis* enhanced resistance to *Verticillium dahlia* but inhibited the growth of rosette leaves [5]. *ARABIDOPSIS SH4-RELATED 3* (*ASR3*) overexpressed plants were smaller than the control but enhanced susceptibility to infections of *Pseudomonas syringae* pv *tomato DC3000* and *Pseudomonas syringae* pv *maculicola ES4326* [6]. Meanwhile, a similar mechanism was also found in the over-accumulation of the ASR3-interacting transcriptional factor 1 (AITF1), which negatively regulated *Pseudomonas syringae* resistance in *Arabidopsis* [7]. In maize, the seedlings of *ZmGT-3b* knockdown showed reduced photosynthesis activity but were resistant to the *Fusarium graminearum* challenge [8]. However, few data verified the role of the trihelix gene in hypertrophy developments. 

Most studies focus on trihelix factor functions in abiotic stress. The overexpression of *ShCIGT* (GT-1) improved cold and drought tolerance in tomatoes [9]. In cotton, *GhGT26* (GT-1)-overexpressed lines had higher salt tolerance than the control via the ABA independent pathway, which was partially similar to the SIP1subfamily gene *GhGT23* [10]. In rice, the experimental data proved that *OsGTgamma-1* and *OsGTgamma-2* have specific roles in promoting salt tolerance when directly regulating salinity transporter genes [11,12]. Interestingly, *SlbHLH22* enhanced plant tolerance to salinity in MicroTom (one dwarf cultivar of tomato) [13]. It was a hypothesis that perhaps *SlbHLH22* regulates abiotic stress-related genes via the trihelix family. 

Aside from regulation by the transcription level, trihelix factor functions are often affected by post-transcription modification. Calcium/calmodulin kinase II (CaMKII) can phosphorylate GT-1 at T133 [14]. ShCIGT (SlGT-24) regulated abiotic tolerance by interacting with Snf1-related kinase 1 (SnRK1) [9]. NMR titration experiments suggested the phosphorylation site of GT-1 is located at the N-terminus of the third helix [15]. The N-terminal of PTL, a GT-2 factor, can be phosphorylated by SnRK1α1(AKIN10), an α-subunit of SnRK1 [16]. Meanwhile, ASR3 can be phosphorylated by MAMP-activated MPK4 [6]. Therefore, we speculated that trihelix factors might fulfill the necessary functions via phosphorylation.

In our experiment, transcriptome and metabolome analysis was used to reveal the molecular mechanism of a developmental malformation in OE, suggesting that the susceptibility of OE plants to *Xanthomonas* was related to increasing salt or oxidant tolerance. Extensive analysis indicated that *GTgamma* was suppressed downstream of SlbHLH22 protein, which was similar to that inhibited expression in salt stress. Deep analysis forecasted that the GTgamma protein might be phosphorylated at the post-transcription level. Therefore, our research provided a good foundation for studying the pathogenic mechanism of hypertrophy development and GTgamma’s role in biotic and abiotic stress.

## 2. Materials and Methods

### 2.1. Plant Materials and Growth Conditions

*Solanum lycopersicum* Mill. var. Ailsa Craig (AC^++^, WT) and *SlbHLH22* (Solyc03g097820, also called *SlUPA-like*) OE lines [3] were grown in a glasshouse under controlled conditions with 16-h-light/8-h-dark cycles, 25 °C-day/18 °C-night temperatures, 80% relative humidity, and 250 μmol m^−2^ s^−1^ luminous intensity. Flowers were tagged at the anthesis stage, immature green fruit was defined as 20 DPA (days past anthesis), mature green fruit as 35 DPA, and breaker fruit as 38 DPA with the color starting to generate a slight yellow shade. Other fruits from the 4th (B+4) and 7th (B+7) days after the breaker were harvested. Fruits at different ripening stages were collected, frozen immediately in liquid nitrogen, and stored at −80 °C until use [17].

### 2.2. Transcriptome and Metabolome Analysis

Total RNA was extracted from OE and WT leaves by using Trizol reagent (Invitrogen, Carlsbad, CA, USA), and the concentration and purity of RNA were measured by Nanodrop 2000 (Thermo Fisher Scientific, Waltham, MA, USA). The RNA integrity was measured by Agient 2100, LabChip GX (Santa Clara, CA, USA). Three biological replicates were sampled for each group (WT, OE). RNA and then transcriptomic experiments were conducted by BMKcloud, Beijing, China (http://www.biomarker.com.cn, accessed on 19 May 2023). Clean reads were obtained by removing adapters. Reads were then mapped to the *Solanaceae* genome (https://solgenomics.net/, accessed on 19 May 2023) using HISAT2 and gene expression levels were quantified with HTseq (BMKcloud, Beijing, China) [18].

Samples were ground to powder using a grinder (MM 400, Retsch, Shanghai, China) and dissolved into an extraction solution to remove by ultrasonic extraction. The extracted metabolites were analyzed by LC-MS/MS with Waters Xevo G2-XS QTOF (Milford, CT, USA). The metabolomics experiments and conjoint analyses of transcriptome and metabolome sequencing were conducted by BMKcloud, Beijing, China (http://www.biomarker.com.cn/, accessed on 19 May 2023) [18].

### 2.3. Hormonal and Salt Treatments

A 35-day-old tomato seedling of AC^++^ planted in green house of Jiujiang University (Jiujiang, China) was used for hormonal and abiotic treatments with three biological replicates. 

For hormonal treatment, all the potted tomato seedlings were sprayed with different hormonal (50 μM 3-Indoleacetic Acid IAA, 50 μM Gibberellin GA, 100 μM 1-Aminocyclopropane-1-Carboxylicacid, ACC, 100 μM Abscisic Acid ABA, 50 μM Methyl Jasmonic Acid MeJA; 50 μM Epibrassinolide EBR; 50 μM Uniconazole NA) (Coolaber, Beijing, Chia) and distilled water (the control). Plants were enclosed in plastic immediately and left for 0, 1, 4, 8, 12, 24 h; the leaves of the tomato seedlings were taken and stored at −80 °C until use [19,20,21].

Salinity treatments were operated by submerging the roots of the tomato seedlings in distilled water with 200 mM NaCl for 0, 1, 4, 8, 12, 24, 48 and 72 h; Roots and leaves from the treated seedlings were collected and stored at −80 °C until use [22].

### 2.4. RT-PCR

The total RNA was reverse-transcribed to cDNA. RT-PCR was performed using SYBR ^®^ Premix Ex Taq TM (TaKaRa, Dalian, China). RT-PCR primers were designed with Primer 5 (Supplementary Table S1). The tomato *SlCAC* and *SlEF1a* gene were used as an internal control of expression patterns and treatments. All the selected genes were calculated with three technical replicates. 

### 2.5. Statistic Analysis

All data are means ± standard deviation of at least three independent experiments. Significance in a difference between the two groups was assessed by a Student’s *t*-test (*, *p* < 0.05 or **, *p* < 0.01). The different letters above the column in the figures indicate that significant differences of *p* < 0.05 were assessed by ANOVA. These statistical programs were performed using DPS v2.1.3 software (Ruifeng, Hangzhou, China).

### 2.6. Computational Modeling

The structure of the peptides was drawn using SWISS-MODEL. The peptide was sent to the GRAMMX protein–protein docking server (Version 12.0). Conformation models were obtained. These docking conformations were sent to the Rosetta FlexPepDock 4.0 server to be refined from a complex between a protein receptor and an estimated conformation for a peptide, allowing full flexibility to the peptide and sidechain of the receptor. FlexPepDock 4.0 gave an output of predicted energies for the complex. Peptides were added to the CHARMM36 force field to correct any resulting mischarges [23].

## 3. Results

### 3.1. Metabolome Analysis of OE vs. WT

**Figure 1:**
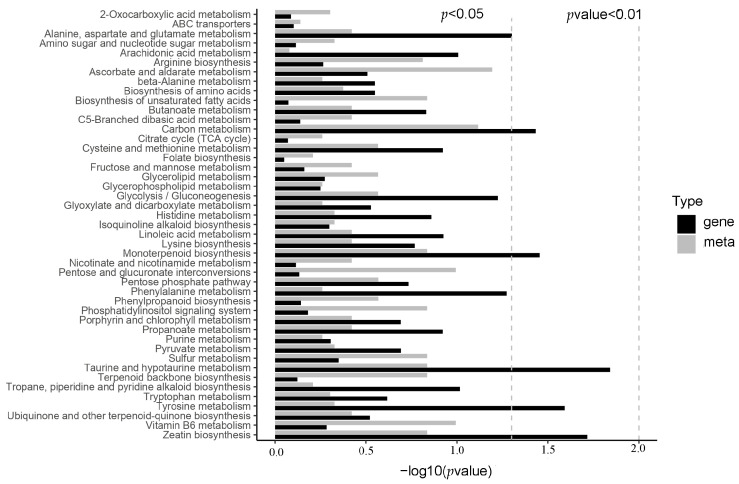
Pathways annotated with differential metabolic process and genes by KEGG analysis. Gene: related to metabolic process; Meta: metabolic process.

After metabolome analysis, different expressed genes (DEGs) encoding metabolic processes in OE were primarily clustered in “alanine, aspartate and glutamate metabolism”, “carbon metabolism”, “monoterpenoid biosynthesis”, “taurine and hypotaurine metabolism”, “tyrosine metabolism” and “zeatin biosynthesis” compared to those in WT. The different metabolic processes were most enriched in “ascorbate and aldarate metabolism”, “carbon metabolism”, “pentose and glucuronate interconversions”, and “vitamin B6 metabolism”. They were further enriched in “arginine biosynthesis”, “unsaturated fatty acids biosynthesis”, “monoterpenoid biosynthesis”, “phosphatidylinositol signaling system”, “sulfur metabolism”, “taurine and hypotaurine metabolism”, “terpenoid backbone biosynthesis”, and “zeatin biosynthesis” (Figure 1). The consistent results between metabolic processes and their DEGs were “carbon metabolism”, “monoterpenoid biosynthesis”, “taurine and hypotaurine metabolism”, and “zeatin biosynthesis”. Within these processes, six key metabolites were abundant, including Acetylserine/O-Acetyl-L-serine (OAS), Glucono-1,5-lactone, Gluconate, 2-Oxoglutarate (2-OG) and Loganate (Figure S1). Previous studies confirmed that these metabolites were helpful to salt or oxidant resistance [24,25,26,27,28].

### 3.2. Transcriptome Analysis of OE vs. WT

**Figure 2:**
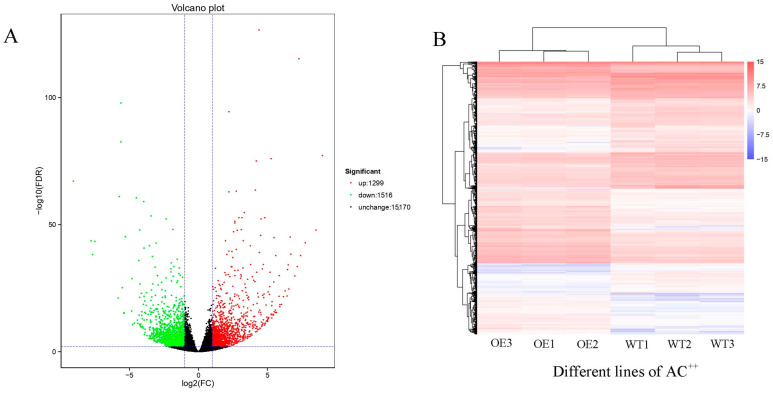
Comparing DEGs by volcano (**A**) and heatmap (**B**) pictures.

To better understand the molecular mechanism of malformation developments in OE leaves, we performed transcriptome analysis in the mature leaves of OE vs. WT. Through RNA-seq analysis, we obtained 6 RNA-seq libraries and 24 to 27 million clean reads. After alignment with reference sequences, the alignment efficiency of clean reads ranged from 94.47% to 96.22% (Supplementary Table S2). Clearly, 2815 DEGs were identified, including 1299 upregulated and 1516 downregulated DEGs (Figure 2). 

**Figure 3:**
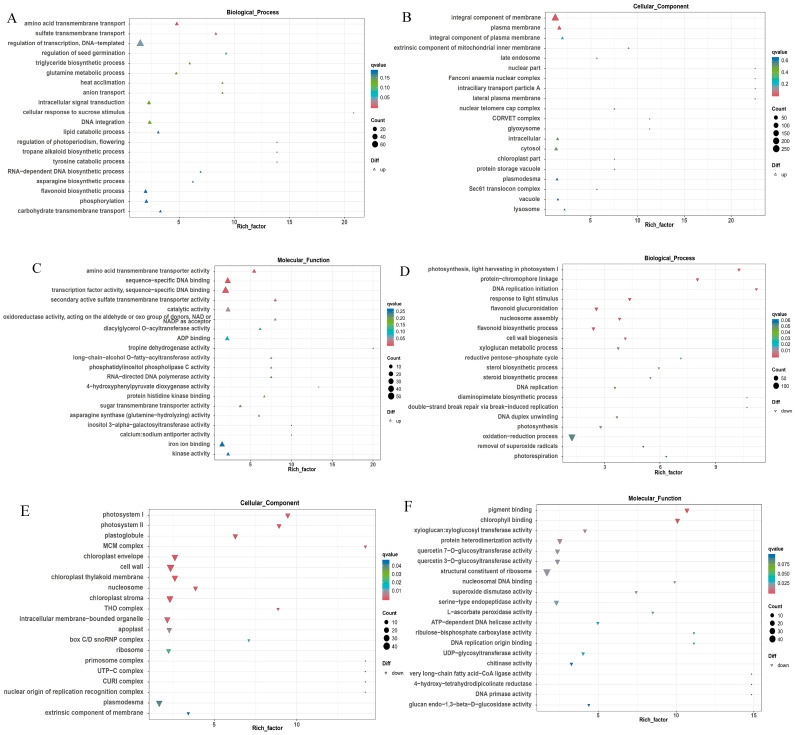
Go enrichment analysis of up—(**A**–**C**) and down—(**D**–**F**) regulated DEGs.

Gene ontology (GO) analysis clarified that upregulated DEGs remarkably converged on “amino acid” and the “sulfate transmembrane transport process” in the biological process (Figure 3A). In cellular component ontology, “integral component of membrane” and “plasma membrane” were the most abundant categories (Figure 3B). Genes involved in “amino acid transmembrane transporter activity”, “sequence-specific DNA binding”, “transcription factor activity” and “secondary sulfate transmembrane transporter activity” were enriched in the molecular function category (Figure 3C). Downregulated DEGs markedly gathered in “photosynthesis”, “light harvesting in PSI”, “protein-chromophore linkage”, “responses to light stimulus”, “flavonoid glucuronidation”, “flavonoid synthesis”, “DNA replication initiation” and “cell wall biogenesis” in biological processes (Figure 3D). In cellular component ontology, “photosystem”, “plastoglobule”, “MCM complex”, “chloroplast”, “cell wall”, “nucleosome”, “intracellular membrane-bounded organelle”, and ”THO complex” were the most abundant categories (Figure 3E). Genes involved in “chlorophyll” and “pigments binding” were enriched in the molecular function category (Figure 3F). These data suggested that the strongly repressed photosynthesis increased the substance transmembrane transport and transcription factor activities in OE. 

**Figure 4:**
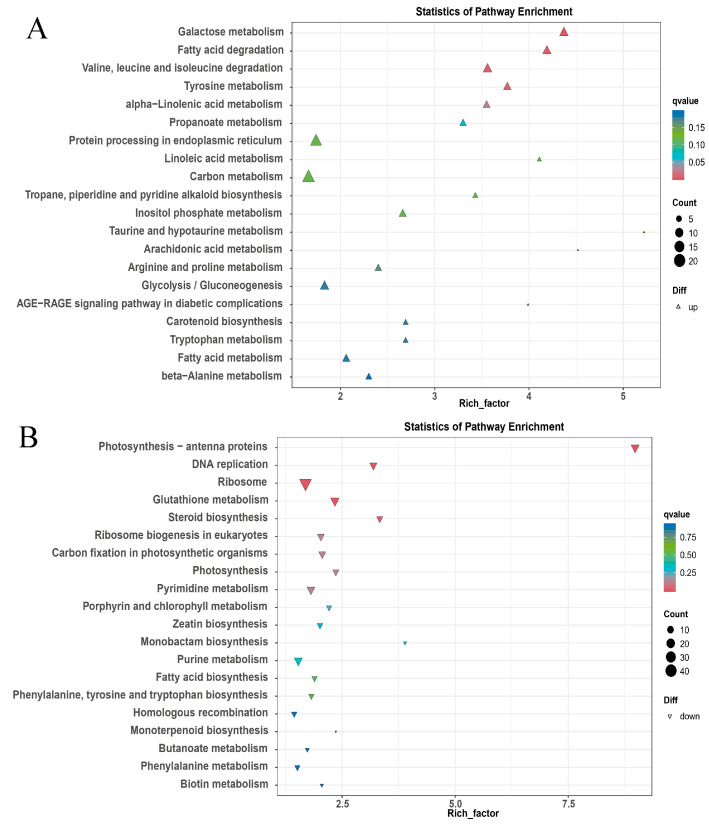
KEGG analysis of up—(**A**) and down—(**B**) regulated DEGs.

Our KEGG enrichment analysis is shown in Figure 4A. The pathway “galactose metabolism”, “fatty acid degradation”, “amino acids (valine, leucine and isoleucine) degradation”, “tyrosine metabolism” and “α-linolenic acid metabolism” were primarily clustered. From a wider range of KEGG enrichment results, “protein processing in endoplasmic reticulum”, “ubiquitin mediated proteolysis”, “plant hormone signal transduction”, and the “phosphatidylinositol signaling process” were also enriched (Figure S2). Downregulated DEGs clustered in “antenna proteins”, “DNA replication”, “ribosome”, “glutathione metabolism”, “steroid biosynthesis” and “ribosome biogenesis” (Figure 4B). These results point to accelerated energy consumption, decreased growth, and development processes in OE.

### 3.3. Analysis of the Transcription Factor among DEGs

**Figure 5:**
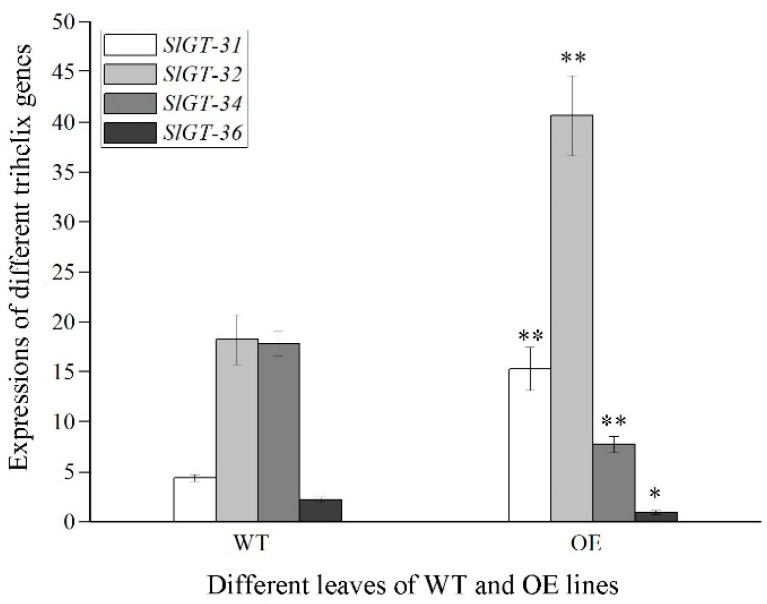
qRT-PCR validation of four differentially expressed trihelix genes in WT vs OE. All data are means ± standard deviation of at least three independent experiments. Significance in difference between the two groups was assessed by a Student’s *t*-test using DPS software (*, *p* < 0.05; **, *p* < 0.01).

GO analysis indicated that DEGs encoding transcription factors were significantly enriched in downstream genes. Through an amino acid blast in the NCBI and SGN databases (plantTFDB), 206 DEGs and 46 TF (transcription factors) families were obtained in OE (Table 1). Trihelix factors always take part in plant photosynthesis, growth, and development [29,30]. Four genes of the trihelix family in OE were clearly regulated, including upregulated *SlGT-31* (GT-2) and *SlGT-32* (SIP1) and downregulated *SlGT-34* (GT-2) and *SlGT-36* (GTgamma) (Figure 5). Recently, the role of the GTgamma subfamily in salt stress has been emphasized [12], but *GTgamma* gene responses in hypertrophy development have rarely been reported.

### 3.4. Expression Patterns of GTgamma Genes in AC^++^ and Their Responses to External Stimuli

**Figure 6:**
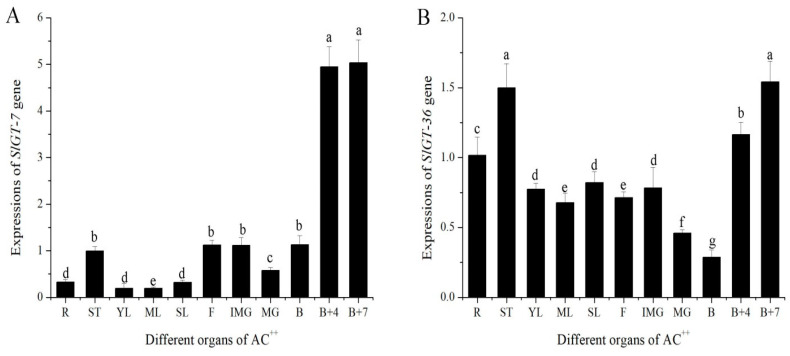
Expressions patterns of *GTgamma* genes, *SlGT-7* (**A**) and *SlGT-36* (**B**) in AC^++^. R: roots; ST: stem; YL: young leaves; ML: mature leaves; SL: senescent leaves; F: flowers; IMG: immature green fruit; mature green fruit; breaker fruit; B+4: 4 days after breaker fruit; B+7: 7 days after breaker fruit; All data are means ± standard deviation of at least three independent experiments. The different letters above the column indicated that significant expressions of *GTgamma* genes among diverse organs were assessed by ANOVA (*p* < 0.05) using DPS software.

Given that *GTgamma* is a downstream gene of SlbHLH22 protein and has a positive function in salt tolerance in rice [12], *GTgamma* responses to salt treatments and expression patterns were investigated in tomatoes. We tested the expression profiles of 11 different organs of the tomato cultivar AC^++^. Two *GTgamma* genes (*SlGT-7* and *SlGT-36*) were expressed in the leaves of AC^++^, especially *SlGT-36*. *SlGT-7* displayed significantly higher expressions in B+4 and B+7 (Figure 6A). *SlGT-36* transcripts accumulated the lowest in the B stage (Figure 6B). Thus, the expression patterns of two *GTgamma* genes exhibited tissue specificity. 

**Figure 7:**
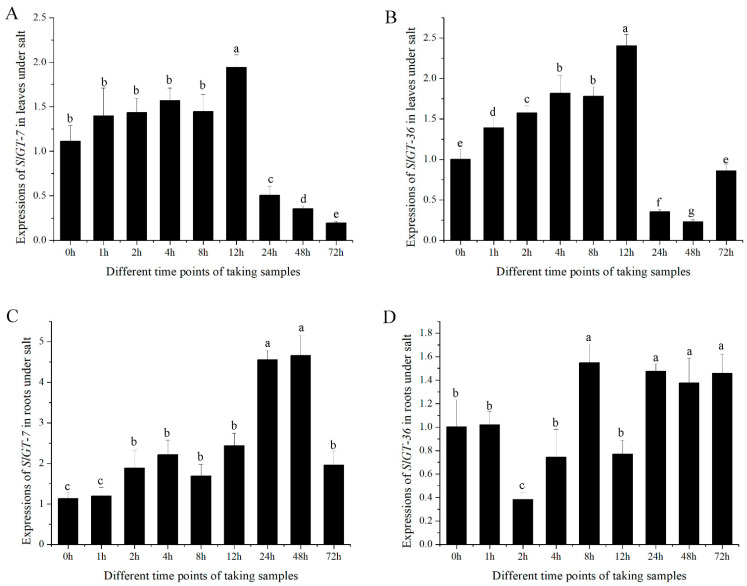
Expressions of *GTgamma* genes, *SlGT-7* (**A**,**C**) and *SlGT-36* (**B**,**D**) in salt stress. The leaves and roots of a 35-day-old AC^++^ seedling were used. All data are means ± standard deviation of at least three independent experiments. The different letters above the column indicate that significant expressions of *GTgamma* genes among diverse time points were assessed by ANOVA (*p* < 0.05) using DPS software.

To examine the endogenous response of *GTgamma* genes to salinity, 35-day-old tomato seedlings were watered with salinity (Figure 7). Both *SlGT-7* and *SlGT-36* were gradually induced to 2~2.5 fold at 12 h and then suddenly suppressed to less than 50% at 24 h in leaves. In the next two days, they remained at a low level (Figure 7A,B). In seedling roots, *SlGT-7* was gradually upregulated to about 4.5-fold within 48 h and then downregulated (Figure 7C,D). The experimental results suggested that both *GTgamma* genes were repressed in leaves due to salinity stress. 

**Figure 8:**
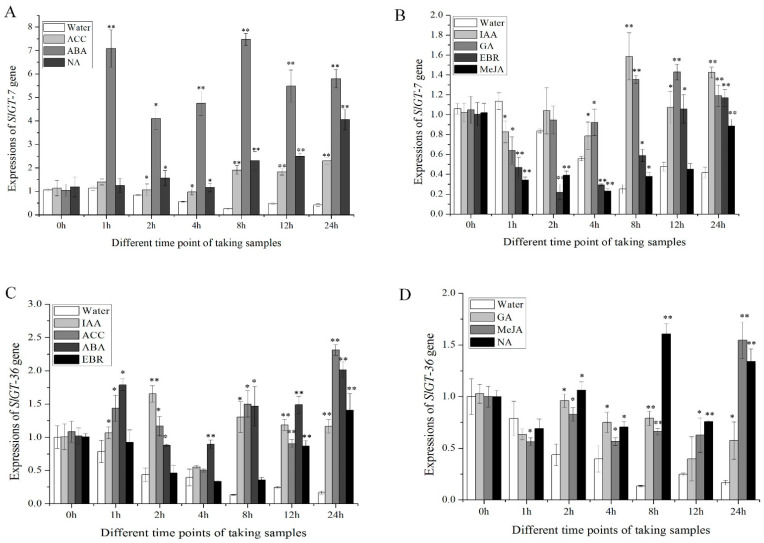
Expressions of two *GTgamma* genes, *SlGT-7* (**A**,**B**) and *SlGT-36* (**C**,**D**), in hormonal treatments. IAA: 3-Indoleacetic Acid; GA: Gibberellin; ACC: 1-Aminocyclopropane-1-Carboxylicacid; ABA: Abscisic Acid; MeJA: Methyl Jasmonic Acid; EBR: Epibrassinolide; NA: Uniconazole. The leaves of 35-day-old AC^++^ seedlings were used. All data are means ± standard deviation of at least three independent experiments. Significance in different expressions of *GTgamma* genes between hormonal treatments and control were assessed by a Student’s *t*-test using DPS software (*, *p* < 0.05; **, *p* < 0.01).

To find the putative signaling pathway, *SlGT-7* and *SlGT-36* were treated with seven hormones. The expression levels of both *GTgamma* genes were higher in all hormonal treatments than in water spraying after 8 h (Figure 8A−D). Within 24 h, *SlGT-7* and *SlGT-36* were maintaining higher levels than controls under ABA treatments (Figure 8A,C). In addition, both *GTgamma* genes showed sensitivity to other hormonal stimuli (Figure 8B,D). These results suggested that *GTgamma* genes might participate in the ABA signaling pathway.

### 3.5. Three-Dimensional Structures of SlGT-7 and Its Potential Phosphorylation Site

**Figure 9:**
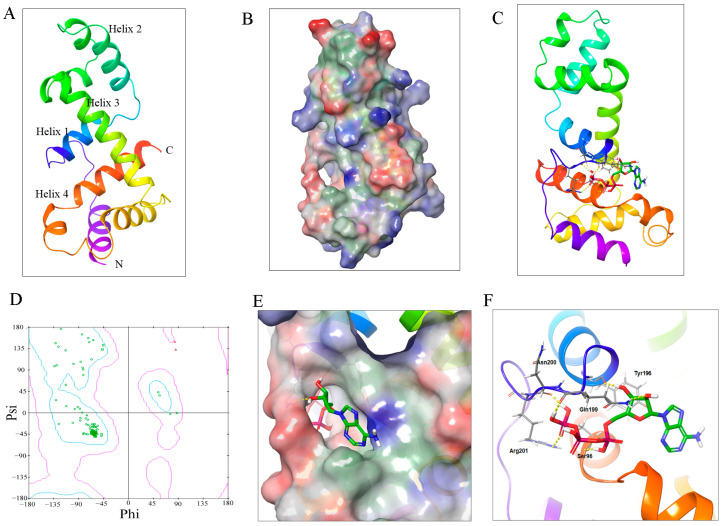
Construction of SlGT-7 model and interaction between SlGT-7 and ATP molecular by autodock. (**A**): The ribbon models of SlGT-7; (**B**): Three-dimensional model of SlGT-7 protein. Electrostatic potential: Positive (blue), negative (red) and hydrophobic (green); (**C**): Interactions of SlGT-7 and ATP molecular as ribbon models; (**D**): Ramachandran plot showing the lowest energy of all the amino acids interacting with the ATP molecular. Phi and Psi represent the rotation angle of the C-N and C-C bonds of α carbon in every peptide unit, respectively. Blue curves indicate the low energy and red the high energy. The dot represents amino acid; (**E**): The putative action site of the SlGT-7 model and ATP molecular; (**F**): The binding of SlGT-7 and ATP by hydrogen bonds.

Transcription factors have a critical role in plant physiology and development, and most of these events are commonly mediated by protein phosphorylation [15,16]. To anticipate the posttranscriptional modification of GTgamma factors, a three-dimensional model of SlGT-7 was built. Using SWISS-MODEL, the lowest energy structure of SlGT-7 is shown as ribbon models in Figure 9A. In this model, two classical domains were found including triple-helix (Helix 1, Helix 2 and Helix 3) and the fourth helix at the C-terminal. SlGT-7 looked like an ellipse with a hole on one side (Figure 9B). ATP molecules putatively entered into the hole and interacted with SlGT-7 at the lowest energy (−6.35 kcal/mol) (Figure 9C–E). Further analysis showed that five amino acids (S^96^, Y^196^, Q^199^, N^200^ and R^201^) inside the hole interacted with the ATP molecules via hydrogen bonds (Figure 9F). The distance estimation of γ-phosphate to five amino acids implied that S^96^ in Helix 1 was the potential phosphorylation site. 

## 4. Discussion

*Xanthomonas* delivers TALes into plant cells to overcome a plant’s defense [1]. Like a transcription factor, AvrBS3, one TALe targets *UPA20* to induce hypertrophy development in pepper leaves, which promotes the infection of *Xanthomonas* [2,3] and *SlUPA-like* (*SlbHLH22*) functions in tomato leaves [2,4]. To reveal the malformation development of OE leaves in more depth, transcriptome and metabolome analyses were carried out in WT vs. OE. The metabolome results showed that the following metabolites were over-accumulated: Acetylserine, O-Acetyl-L-serine (OAS), Glucono-1,5-lactone, Gluconate, 2-Oxoglutarate (2-OG), and Loganate (Figure 1). OAS accumulations are related to resistance to salt stress [24,25], which was analogous to the biological function of the GTgamma factor in rice [12]. Gluconate induces increased abiotic stress resistance in plants [28]. 2-OG is linked to the metal toxicity alleviatory of tomato and hormonal synthesis in the sulfate-dependent or independent pathway [26,31], which was similar to our results in the GO analysis (Figure 3). Through RNA-seq analysis, 1299 and 1516 DEGs were, respectively, up- and downregulated (Figure 2). The transcriptome enrichment results indicated that weak photosynthesis, high-energy consumption, increased transcription factor activity, and sulfate transmembrane transport occurred in OE (Figure 3 and Figure 4). Loganate has the capability of scavenging against superoxide radicals [25]. In addition, *SlbHLH22* (also called *SlUPA-like*) enhances plant salinity [13,32]. Therefore, both transcriptome and metabolome analyses suggested that the hypertrophy phenotypes of OE lines might be connected with promoting salt or oxidative resistance. 

Further research showed that the *GTgamma* gene was not only suppressed in hypertrophy leaves, but also inhibited by salt stress. The GO analysis showed that these biological processes, e.g., “light harvesting”, “photosynthesis”, “responses to light stimulus”, “flavonoid synthesis”, etc., were prominently restrained in OE, which always took place in the trihelix factor [30,33]. Fortunately, four trihelix genes exhibited remarkable regulation: increased *SlGT-31*(GT-2) and *SlGT-32* (SIP1) and decreased *SlGT-34* (GT-2) and *SlGT-36* (GTgamma) (Figure 5). Furthermore, six metabolites (Acetylserine, OAS, Glucono-1,5-lactone, Gluconate, 2-OG and Loganate) had a possible role in promoting salt or oxidant tolerance [24,25,26,27,28]. It was reported that GTgamma played the role of a positive regulator in salt stress in rice and that *SlbHLH22* boosted salt resistance in tomatoes [11,12,13]. These results implied that *GTgamma,* as downstream genes of SlbHLH22 protein, might perform a salt-resistant function in tomatoes. Figure 7 shows that both *GTgamma* genes were prominently inhibited by salt stress, implying a consistent role in malformation development of the OE line and salt stress.

Through an extensive analysis of the *GTgamma* genes, we found that two *GTgamma* genes were expressed in AC^++^ leaves, especially *SlGT-36*, indicating the reason why only one *GTgamma* gene was repressed by *SlbHLH22* in hypertrophy. Tissue-specific expression patterns were present when *SlGT-7* transcripts were specifically expressed in B+4 and B+7 stages fruit and *SlGT-36* in all tissues except B stage fruit (Figure 6), which was slightly different from Yu et al. [34], indicating the following different varieties: AC^++^ and LA1777. In addition, *SlGT-7* was remarkably upregulated by ABA, which was very similar to *OsGTgamma-1* [11]. Both *SlGT-7* and *SlGT-36* responded to all selected phytohormone, indicating their versatile role in plant growth and development (Figure 8). Moreover, we also found that water inhibited *SlGT-7* and *SlGT-36* expressions by over 60% in the leaves of AC^++^ seedlings (Figure 8). Whether SlGT-7 was involved in the regulation of water stress needs more evidence. 

Protein posttranslational modification is a fine-tuned mechanism in abiotic or biotic resistance [6,9,15,16,17]. Therefore, we hypothesized that GTgamma performed this function via phosphorylation but required further experimental evidence support. We constructed a three-dimensional model of SlGT-7 as a candidate. We discovered the interactions between ATP and SlGT-7 in a putative hole (Figure 9). We also predicted that S^96^ was the most likely phosphorylation site. It was commonly believed that protein kinases transfer γ-phosphate from ATP to Ser (S), Thr (T), or Tyr (Y) during protein modification [35]. Our model implied that S^96^ got closer to the γ-phosphate of ATP than others, suggesting the phosphorylation site of S^96^ (Figure 9F). In short, our present findings about the posttranslational modification model of the GTgamma protein provide the foundation for an in-depth study of the hypertrophy development of OE lines and the regulatory role of downstream genes in tomatoes.

## 5. Conclusions

*Xanthomonas* injects TALes into the host cells to suppress plant immune defense. One TALe, AvrBS3, activates the plant target gene: pepper *upa20*. The overexpression of *SlbHLH22* (also called *SlUPA-like*), i.e., the orthology of *upa20*, causes the hypertrophy and susceptibility of *Xanthomonas* in tomatoes. The metabolome analysis showed that specific metabolites were over-accumulated in OE with a potential role in promoting salt resistance. The transcriptome analysis verified that OE plants suffered from high energy consumption, weak photosynthesis, and increased transcription factors activity. *GTgamma* gene expression was suppressed by *SlbHLH22*. Furthermore, it was simultaneously inhibited by salt stress, indicating GTgamma’s role in the formation of hypertrophy development via the salt stress response. Extensive analysis proved that both *GTgamma* genes expressed in leaves were induced by ABA. Moreover, the GTgamma protein had a putative phosphorylation site at S^96^. Our results provide the basis for disclosing the pathogenic mechanism of hypertrophy development medicated by the GTgamma subfamily.

## Figures and Tables

**Table 1:** Statistical analysis of all differentially expressed transcription factor genes.

Serial Number	TF Family	DEGs Numbers	Serial Number	TF Family	DEGs Numbers	Serial Number	TF Family	DEGs Numbers
1	AP2/ERF-AP2	2	17	E2F-DP	1	33	MYB-related	4
2	AP2/ERF-ERF	19	18	EIL	1	34	NAC	19
3	B3	5	19	GARP-ARR-B	1	35	NF-YA	4
4	B3-ARF	2	20	GARP-G2-like	2	36	NF-YB	1
5	BBR-BPC	1	21	GeBP	1	37	NF-YC	1
6	bHLH	15	22	GRAS	4	38	OFP	1
7	bZIP	10	23	HB-BELL	2	39	PLATZ	2
8	C2C2-CO-like	2	24	HB-HD-ZIP	15	40	RWP-RK	1
9	C2C2-Dof	5	25	HB-KNOX	2	41	SRS	1
10	C2C2-GATA	3	26	HB-other	4	42	TCP	6
11	C2C2-YABBY	2	27	HMG	2	43	Tify	2
12	C2H2	11	28	HSF	6	44	Trihelix	4
13	C3H	2	29	LOB	1	45	WRKY	8
14	CPP	1	30	MADS-MIKC	8	46	zf-HD	1
15	DBB	1	31	MADS-M-type	3			
16	DBP	1	32	MYB	16			

## Data Availability

The data presented in this study are available within the article and the Supplementary Materials.

## Supplementary Materials

The following supporting information can be downloaded at: https://www.mdpi.com/article/10.3390/metabo13121195/s1, Table S1: The primers used for qRT-PCR analysis; Table S2: Quality control of RNA-seq reads in different samples; Figure S1: Key metabolites in four metabolic processes; Figure S2: Comprehensive analysis of up—(A) and down—(B) regulated DEGs by KEGG.
