# Supplementary material for: SlbHLH22-Induced Hypertrophy Development Is Related to the Salt Stress Response of the GTgamma Gene in Tomatoes
Source: Metabolites. 2023 Dec 11;13(12):1195. doi: 10.3390/metabo13121195 (PMC10744757; doi:10.3390/metabo13121195)
Supplement: Supplementary file 1 [file metabolites-13-01195-s001.zip › metabolites-2720660-supplementary.pdf]

metabolites

Supplementary Materials

SlbHLH22–Induced Hypertrophy Development Is Related to the Salt Stress Response of the *GTgamma* Gene in Tomatoes

Baolu Cui [1,2], Min Yu [1], Jiaojiao Bai [1,*] and Zhiguo Zhu [1,*]

1 College of Pharmacy and Life Sciences, Jiujiang University, Jiujiang 332005 China; 6090111@jju.edu.cn (B.C.); yuminyumin@whu.edu.cn (M.Y.)
2 College of Biological Sciences and Agriculture, Qiannan Normal University for Nationalities, Duyun 558000, China
* Correspondence: jjbai@sjziam.ac.cn (J.B.); zhuzhiguo@jju.edu.cn (Z.Z.)

Supplementary Information

Table S1. The primers used for qRT-PCR analysis.

Primer Name	Forward Sequences（5'→3'）	Reverse Sequences（5'→3'）
SlCAC	CCTCCGTTGTGATGTAACTGG	ATTGGTGGAAAGTAACATCATCG
SlEF1a	TACTGGTGGTTTTGAAGCTG	AACTTCCTTCACGATTTCATCATA
SlGT-7	TGGGAGAGGTACGGCATGTA	AATCCTGCGACATGCTTCCA
SlGT-31	GCACAAGAAGTACCCCTCCC	CAAGGAAAGCAATAAGAGCCAA
SlGT-32	TCGTCGCCAGTCCATCCT	TTCTTCGTGTTGCCGTGA
SlGT-34	ACCATCAAGCTTCTAAGCTATCCG	TTTAAACTCCTCTTCCCCTCCTC
SlGT-36	GTCCTGGAGTGGGGTTGAAG	GCACAAGAAGTACCCCTCCC

Table S2. Quality control of RNA-seq Reads in different samples.

Samples	Total Reads	Clean Reads	Mapped Reads	Unique Mapped Reads	≥Q30
WT1	48,083,964	24,041,982	45,901,481 (95.46%)	44,645,294 (92.85%)	95.46%
WT2	45,174,334	22,587,167	43,276,087 (95.80%)	42,311,051 (93.66%)	95.55%
WT3	46,112,196	23,056,098	44,245,580 (95.95%)	43,260,602 (93.82%)	95.25%
OE1	55,403,172	27,701,586	53,308,306 (96.22%)	51,967,013 (93.80%)	95.24%
OE2	45,701,750	22,850,875	43,175,875 (94.47%)	42,020,606 (91.95%)	95.15%
OE3	55,210,616	27,605,308	53,059,572 (96.10%)	51,141,744 (92.63%)	95.14%

A

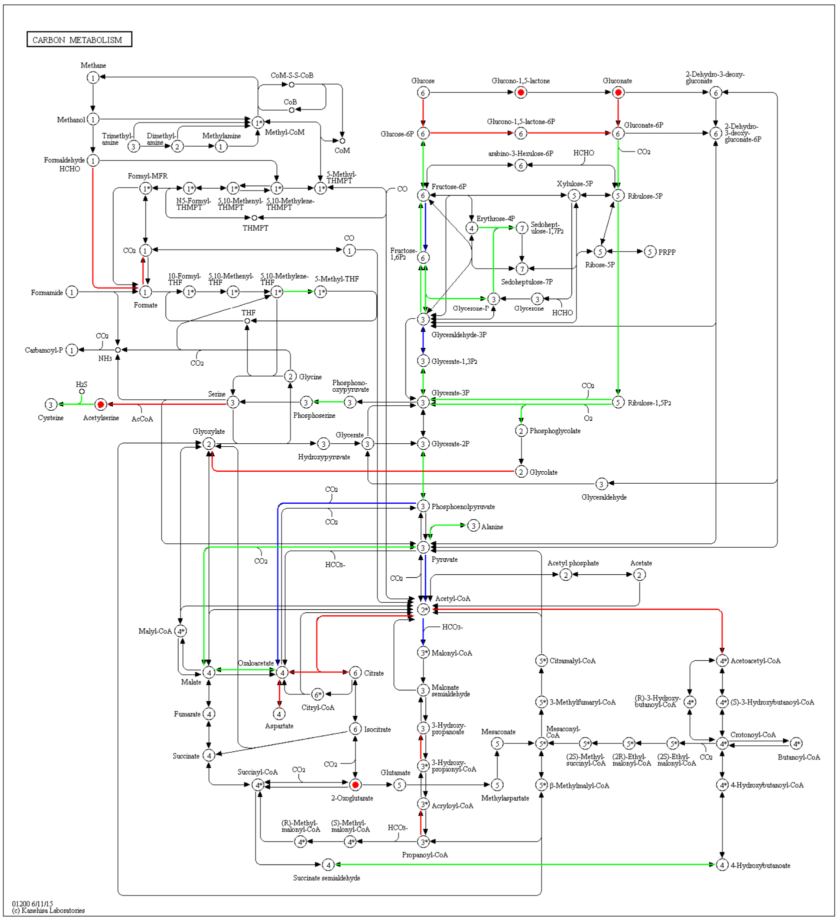
CARBON METABOLISM
Methane
CoM-S-S-CoB
CoB
Methanol
Trimethyl-amine
Dimethyl-amine
Methylamine
Methyl-CoM
CoM
Formaldehyde HCHO
Formyl-MFR
N5-Formyl-THMPT
5,10-Methenyl-THMPT
5,10-Methylene-THMPT
5-Methyl-THMPT
THMPT
CO
CO2
10-Formyl-THF
5,10-Methenyl-THF
5,10-Methylene-THF
5-Methyl-THF
Formamide
Formate
THF
Carbamoyl-P
NH3
Glycine
Serine
Phosphono-oxypyruvate
Phosphoserine
H2S
Cysteine
Acetylserine
AcCoA
Glyoxylate
Glycerate
Hydroxypyruvate
Glucose
Glucono-1,5-lactone
Gluconate
2-Dehydro-3-deoxy-gluconate
Glucose-6P
Glucono-1,5-lactone-6P
Gluconate-6P
2-Dehydro-3-deoxy-gluconate-6P
arabino-3-Hexulose-6P
HCHO
Fructose-6P
Xylulose-5P
Ribulose-5P
Erythrose-4P
Sedoheptulose-1,7P2
Fructose-1,6P2
Ribose-5P
PRPP
Sedoheptulose-7P
Glycerone-P
Glycerone
Glyceraldehyde-3P
Glycerate-1,3P2
Glycerate-3P
Ribulose-1,5P2
O2
Phosphoglycolate
Glycerate-2P
Glycolate
Glyceraldehyde
Phosphoenolpyruvate
Alanine
Pyruvate
HCO3-
Acetyl phosphate
Acetate
Acetyl-CoA
Malyl-CoA
Malonyl-CoA
Malate
Oxaloacetate
Citrate
Citryl-CoA
Aspartate
Malonate semialdehyde
Fumarate
Isocitrate
3-Hydroxy-propanoate
Succinate
Succinyl-CoA
2-Oxoglutarate
Glutamate
3-Hydroxy-propanoyl-CoA
Acryloyl-CoA
Propanoyl-CoA
(R)-Methyl-malonyl-CoA
(S)-Methyl-malonyl-CoA
Succinate semialdehyde
Mesaconate
Methylaspartate
Citramalyl-CoA
3-Methylfumaryl-CoA
Mesaconyl-CoA
β-Methylmalyl-CoA
(2S)-Methyl-succinyl-CoA
(2R)-Ethyl-malonyl-CoA
(2S)-Ethyl-malonyl-CoA
Acetoacetyl-CoA
(R)-3-Hydroxy-butanoyl-CoA
(S)-3-Hydroxybutanoyl-CoA
Crotonoyl-CoA
Butanoyl-CoA
4-Hydroxybutanoyl-CoA
4-Hydroxybutanoate
01200 6/11/15
(c) Kanehisa Laboratories

B

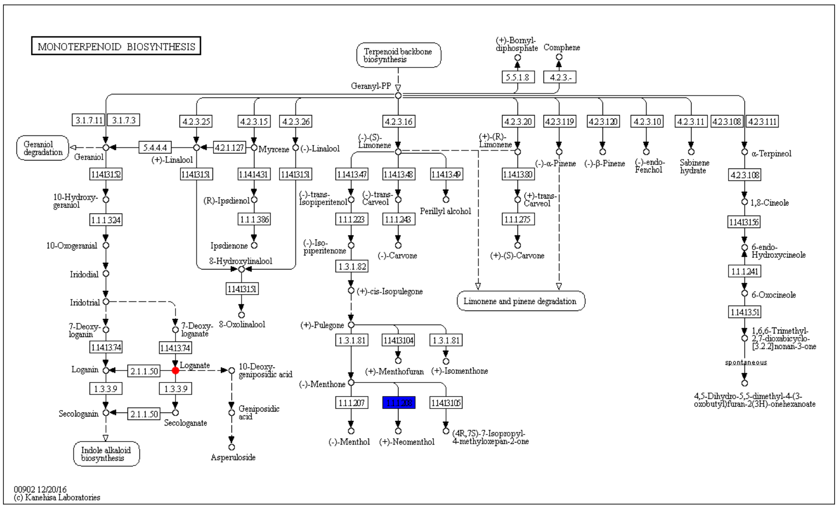
MONOTERPENOID BIOSYNTHESIS
Terpenoid backbone biosynthesis
(+)-Bornyl-diphosphate
Camphene
5.5.1.8
4.2.3.-
Geranyl-PP
3.1.7.11
3.1.7.3
4.2.3.25
4.2.3.15
4.2.3.26
4.2.3.16
4.2.3.20
4.2.3.119
4.2.3.120
4.2.3.10
4.2.3.11
4.2.3.108
4.2.3.111
Geraniol degradation
Geraniol
5.4.4.4
(+)-Linalool
4.2.1.127
Myrcene
(-)-Linalool
(-)-(S)-Limonene
(+)-(R)-Limonene
(-)-α-Pinene
(-)-β-Pinene
(-)-endo-Fenchol
Sabinene hydrate
α-Terpineol
1.14.13.152
1.14.13.151
1.14.14.31
1.14.13.47
1.14.13.48
1.14.13.49
1.14.13.80
4.2.3.108
10-Hydroxy-geraniol
(R)-Ipsdienol
(-)-trans-Isopiperitenol
(-)-trans-Carveol
Perillyl alcohol
(+)-trans-Carveol
1,8-Cineole
1.1.1.324
1.1.1.386
1.1.1.223
1.1.1.243
1.1.1.275
1.14.13.156
10-Oxogeranial
Ipsdienone
(-)-Iso-piperitenone
(-)-Carvone
(+)-(S)-Carvone
6-endo-Hydroxycineole
Iridodial
8-Hydroxylinalool
1.3.1.82
1.1.1.241
Iridotrial
1.14.13.151
(+)-cis-Isopulegone
Limonene and pinene degradation
6-Oxocineole
7-Deoxy-loganin
7-Deoxy-loganate
8-Oxolinalool
(+)-Pulegone
1.14.13.51
1.14.13.74
1.14.13.74
1.3.1.81
1.14.13.104
1.3.1.81
1,6,6-Trimethyl-2,7-dioxabicyclo-[3.2.2]nonan-3-one
Loganin
2.1.1.50
Loganate
10-Deoxy-geniposidic acid
(+)-Menthofuran
(+)-Isomenthone
spontaneous
1.3.3.9
1.3.3.9
(-)-Menthone
Geniposidic acid
1.1.1.207
1.14.13.105
4,5-Dihydro-5,5-dimethyl-4-(3-oxobutyl)furan-2(3H)-onehexanoate
Secologanin
2.1.1.50
Secologanate
(-)-Menthol
(+)-Neomenthol
(4R,7S)-7-Isopropyl-4-methyloxepan-2-one
Indole alkaloid biosynthesis
Asperuloside
00902 12/20/16
(c) Kanehisa Laboratories

C

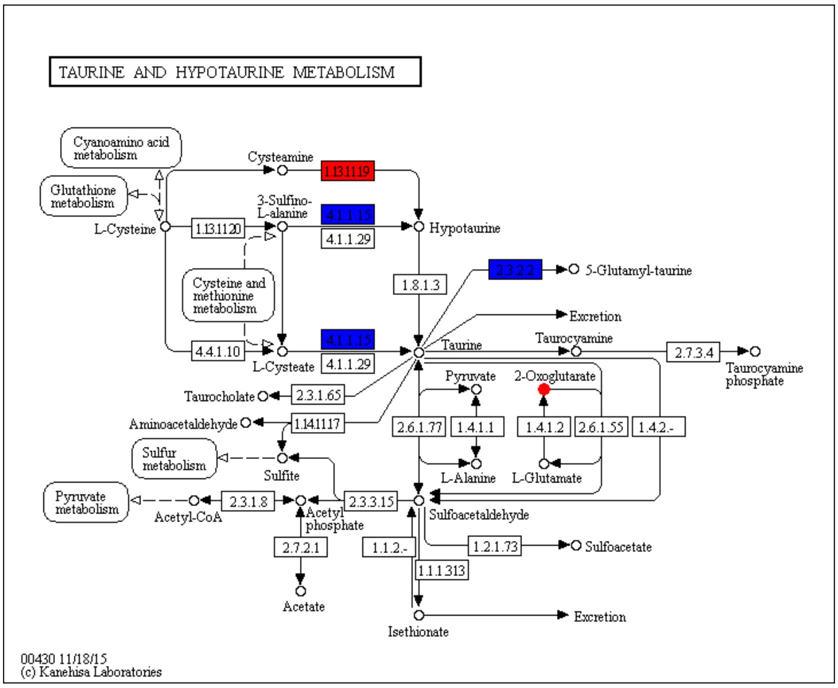

D

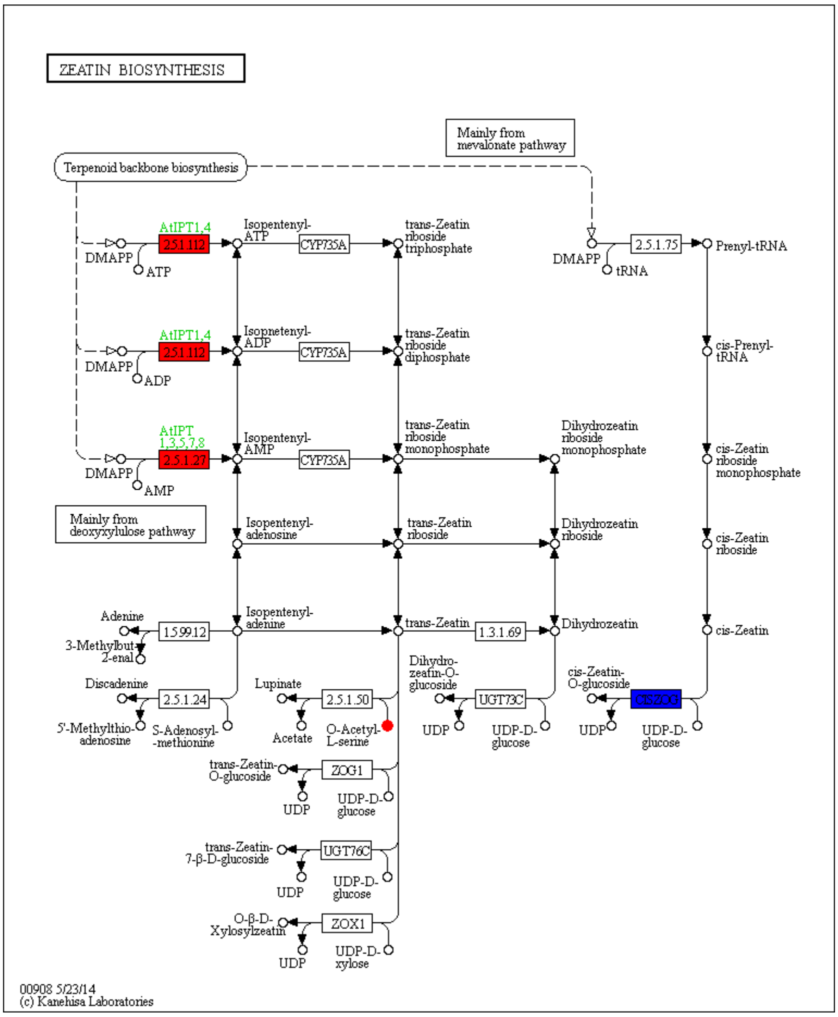

Figure S1: Key metabolites in four metabolic processes. A, carbon metabolism; B, monoterpenoid biosynthesis; C, taurine and hypotaurine metabolism; D, zeatin biosynthesis

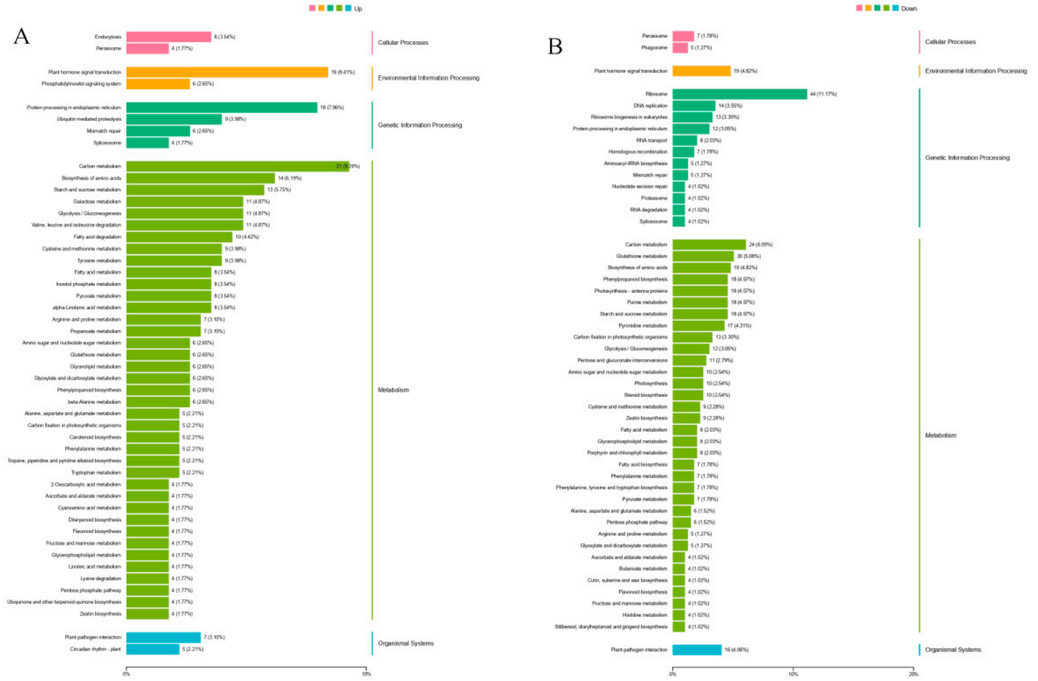

Figure S2. Comprehensive analysis of up- (A) and down-(B) regulated DEGs by KEGG.
